# Extracellular release of virulence factor major surface protease *via* exosomes in *Leishmania infantum* promastigotes

**DOI:** 10.1186/s13071-018-2937-y

**Published:** 2018-06-19

**Authors:** Skye Marshall, Patrick H. Kelly, Brajesh K. Singh, R. Marshall Pope, Peter Kim, Bayan Zhanbolat, Mary E. Wilson, Chaoqun Yao

**Affiliations:** 1 Department of Biomedical Sciences and One Health Center for Zoonoses and Tropical Veterinary Medicine, Ross University School of Veterinary Medicine, St. Kitts & Nevis, West Indies, USA; 2 Department of Microbiology, University of Iowa, Iowa City, IA USA; 3 Department of Internal Medicine, University of Iowa, Iowa City, IA USA; 4 Present address: Stead Family Department of Pediatrics, Carver College of Medicine, University of Iowa, Iowa City, IA USA; 5 The Proteomics Facility, University of Iowa, Iowa City, IA USA; 6 Carver College of Medicine, University of Iowa, Iowa City, IA USA; 7 Department of Epidemiology, University of Iowa, Iowa City, IA USA; 8 Iowa City VA Medical Center, Iowa City, IA USA

**Keywords:** *Leishmania*, Exosome, Major surface protease, Virulence factors, Promastigotes

## Abstract

**Background:**

The *Leishmania* spp. protozoa are introduced into humans through a sand fly blood meal, depositing the infectious metacyclic promastigote form of the parasite into human skin. Parasites enter a variety of host cells, although a majority are found in macrophages where they replicate intracellularly during chronic leishmaniasis. Symptomatic leishmaniasis causes considerable human morbidity in endemic regions. The *Leishmania* spp. evade host microbicidal mechanisms partially through virulence-associated proteins such as the major surface protease (MSP or GP63), to inactivate immune factors in the host environment. MSP is a metalloprotease encoded by a tandem array of genes belonging to three *msp* gene classes, whose mRNAs are differentially expressed in different life stages of the parasite. Like other cells, *Leishmania* spp. release small membrane-bound vesicles called exosomes into their environment. The purpose of this study was to detect MSP proteins in exosomal vesicles of *Leishmania* spp. protozoa.

**Methods:**

Using mass spectrometry data we determined the profile of MSP class proteins released in *L. infantum* exosomes derived from promastigotes in their avirulent procyclic (logarithmic) stage and virulent stationary and metacyclic stages. MSP protein isoforms belonging to each of the three *msp* gene classes could be identified by unique peptides.

**Results:**

Metacyclic promastigote exosomes contained the highest, and logarithmic exosomes had the lowest abundance of total MSP. Among the MSP classes, MSPC class had the greatest variety of isoforms, but was least abundant in all exosomes. Nonetheless, all MSP classes were present at higher levels in exosomes released from stationary or metacyclic promastigotes than logarithmic promastigotes.

**Conclusions:**

The data suggest the efficiency of exosome release may be more important than the identity of MSP isoform in determining the MSP content of *Leishmania* spp. exosomes.

**Electronic supplementary material:**

The online version of this article (10.1186/s13071-018-2937-y) contains supplementary material, which is available to authorized users.

## Background

The *Leishmania* spp. protozoa are the etiological agents of leishmaniasis, a spectrum of diseases causing an estimated 1.4 million new infections annually in 90 countries in five continents [1]. The protozoa are taken up and replicate inside phagocytic cells in humans and can result in either asymptomatic infection or symptomatic leishmaniasis. The most severe form of the disease is visceral leishmaniasis (VL) caused by *Leishmania infantum* or *L. donovani*, during which macrophages of reticuloendothelial organs harbor intracellular parasites. Treatment modalities for VL cause significant toxicity, and the long-standing first-line medicine, i.e. pentavalent antimonials, cannot be used in some regions due to significant parasite resistance [2, 3]. Despite progress in drug development, there are deaths despite adequate therapy [4]. There is a continuing need to study the pathogenic mechanisms employed by the *Leishmania* parasite in an effort to discover novel therapeutic approaches.

The extracellular promastigote of *Leishmania* spp. displays an abundant major surface protease (MSP, also called GP63), a virulence factor that promotes parasite survival both intracellularly, through modulating macrophage killing mechanisms, and extracellularly by evading microbicidal proteins [5]. The process of parasite differentiation in the sand fly vector from procyclic to metacyclic forms is pre-adaptive to infection of a mammal host [6]. MSP protein is differentially regulated in concert with parasite development in the insect. Studies of *L. infantum* show that the abundance of MSP increases approximately 14-fold while the parasite develops from a non-infectious logarithmic growth stage to an infectious metacyclic stage in vitro [7].

The sequences of *msp* genes of *L. infantum* allow them to be divided into three classes, i.e. log (*mspL*), stationary (*mspS*), and constitutive (*mspC*) [8], named according to the in vitro growth stage in which their mRNA is predominantly expressed, and differentiated by variations in nucleotide sequences in their 3' UTRs, with some corresponding changes in the protein-coding regions [9]. The temporal correlation between *msp* gene class expressed and MSP isoform(s) or function in the parasite life-cycle is incompletely understood. It seems likely that the MSPs upregulated in the metacyclic stage might help prepare the parasite for mammalian infection.

It has been shown years ago that MSPs are released in membrane-bound vesicles into the parasite’s extracellular environment [7], and that released MSP promotes migration through extracellular matrix [10]. Exosomes are small vesicles released by many cell types including the *Leishmania* spp., and can facilitate inter-cellular communication [11]. Functionally, *Leishmania* exosomes have been found to be anti-inflammatory and play a role in pathogenesis by modulating the cytokines expressed at the local site of infection [12]. MSP has been previously identified in exosomes released by *L. donovani*, *L. mexicana*, *L. major* and *L. infantum* [12–14].

Recognizing the likely importance of exosomes and of MSPs in pathogenesis of leishmaniasis, the purpose of this study was to compare the abundance and the isoforms of MSP in exosomes released by *L. infantum* promastigotes in their different life-cycle stages, including the non-infective logarithmic and the infective stationary growth stages, and from infectious metacyclic promastigotes purified from stationary *L. infantum* promastigotes. We took advantage of a comprehensive mass spectrometry study of total exosomes proteins released from different developmental stages of *L. infantum* promastigotes (Singh, Kelly, Pope and Wilson, manuscript under review). These data were analyzed specifically to determine the *msp* class gene products in exosomes released by different parasite life-cycle stages, and to draw inferences about the relationship between released products of *msp* genes and parasite virulence.

## Methods

### Parasites and culture

A Brazilian strain of *L. infantum* (MHOM/BR/00/1669) was maintained in male Syrian hamsters by serial passage. Amastigotes were isolated from hamster spleens and incubated in hemoflagellate-modified minimal essential medium (HOMEM) with 10% fetal calf serum (FCS) to allow stage conversion and growth as promastigotes [15]. Promastigotes were used within 3 passages in vitro, since they lose virulence in liquid culture. Promastigote cultures were seeded at 1 × 10^6^ parasites/ml in HOMEM and cultured at 26 °C for 3–8 days to collect logarithmic or stationary growth phases, respectively, as described [16]. Metacyclic promastigotes were separated from stationary phase cultures according to density on a discontinuous Ficoll gradient [17].

### Exosome isolation

Promastigotes were suspended in serum-free growth medium (SFM) [7] at a cell density of 2 × 10^8^ cells/ml and incubated at 26 °C overnight. SFM is similar to HOMEM but lacks FCS and is exosome-free. After centrifugation at 1200× *g* for 10 min, lysates for gel electrophoresis were prepared from the pellet containing promastigotes. The supernatant containing secreted nanovesicles was filtered (0.2 μm steriflip vacuum filter, Millipore, Burlington, USA) to remove residual cells and debris, and nanovesicles were separated from low Mr components with a 100 kDa MWCO Centricon Plus-70 (Millipore). Concentrated vesicles were suspended in 3 ml phosphate-buffered saline (PBS) and washed twice by ultracentrifugation at 110,000× *g* at 4 °C, for four h and 16 h (Beckman Optima L 100 Tabletop Ultracentrifuge, TLA-100.3 rotor, Beckman Coulter Inc., Brea, USA). For LC-MS/MS the pellet was purified for exosomes via flotation in a linear sucrose gradient as previously reported [18].

### Transmission electron microscopy

Washed exosomes were fixed in 2.5% glutaraldehyde in 0.1M Na cacodylate buffer. The exosome suspension was applied to a formvar and carbon covered grid and incubated in 1% ammonium molybdate. After drying, grids were imaged on A Jeol JEM-1230 transmission electron microscope (TEM) (Joel USA Inc., Peabody, USA).

### Immunoblot analysis

Protein concentrations were measured with a Pierce™ BCA protein assay kit (Thermo Scientific, Waltham, USA). Seven μg of exosomes or lysates were suspended in 2 μl of Benzonase, 1 μl of Merck Protease Inhibitor cocktail (Merck, Burlington, USA) in reducing SDS sample buffer, heated (100 °C, 6 min), and separated on 10% Precise Tris-Glycine precast gels (BioRad, Hercules, USA). Duplicate gels were subjected to either immunoblotting or silver stain (Pierce® Silver Stain Kit, Thermo Scientific). For immunoblotting, proteins were transferred on to nitrocellulose filter, blocked in 5% milk, 0.1% Tween 20 in PBS, and probed with a polyclonal sheep anti-MSP diluted 1:10,000 followed by HRP conjugated rabbit anti-sheep IgG (Pierce) (1:10,000 dilution). The sheep anti-MSP polyclonal serum was raised against purified total *L. chagasi* MSP and detected more than 10 different MSPs distributed between *pI*s of 5.2–6.1 and masses of 58–63 kDa in 2-D western blot as previously determined [16, 19].

### LC-MS/MS

The current study was a sub-analysis of a larger study characterizing exosomes from different *L. infantum * growth stages (manuscript under review, Singh, Kelly, Pope and Wilson). Gel slices were subjected to in-gel tryptic digestion as described [20]. LC-MS/MS was performed at the University of Iowa Proteomics Facility. Briefly, peptide samples were desalted with a Dionex 3000 UHP nanoRSLC series HPLC system (Thermo-Electron, Waltham, USA) [21], separated by liquid chromatography (Halo particles with 300 Angstrom pore size, Advanced Material Designs, Huntsville, USA) and directed to a linear ion trap mass spectrometer (Thermo LTQ/XL, Thermo-Electron, Waltham, USA). Raw peptide datasets were combined into a single list using Distiller (version 2.4, MatrixScience, Cambridge, UK). The MASCOT 2.4 database search engine (Matrix Science, Cambridge, UK) was used to search the SwissProt and TremBl databases for protein identification (July 23rd, 2012) [22].

Analysis of the proteomic data from the exosomes was carried out using Scaffold 4.4.1. The requirements for protein isoform identification were a minimum of two unique peptides, with protein and peptide confidence thresholds of 95%. Data were compared to protein sequences from NCBI or UniProt using ClustalW2 Multiple Sequence Alignment program [23]. Relative peptide abundance was determined in comparison to normalized total spectrum counts for all proteins in each sample, calculated by the Scaffold software (ProteomeSoftware, Portland, USA). This allowed us to estimate relative abundances of peptides between several samples, but does not allow for absolute quantification. The normalization algorithm assumes that the total protein loaded in each sample is equal, an assumption that was reasonable since each sample consisted of 5 μg of protein from the isolated exosome preparation (https://www.dropbox.com/s/pxcvfi7wwz4g309/scaffold_qplus_normalization.pdf?dl=1). The normalized total spectrum count (spectral value) was used throughout this study to measure relative quantitative differences.

### Classification of exosome MSP

Two approaches were used to identify a class for each MSP protein. First, protein information about the MSP class was sought on the NCBI or UniProt databases. Secondly, peptides that distinguished a specific class or a subset of classes were identified manually. Most MSP proteins in the databases had not already been assigned to one of the 3 classes; therefore, the latter route was used most often. Some unique peptides useful for distinguishing classes were already known identifiers of MSPS or MSPC classes, and one peptide was unique to MSPL1 [8, 9, 24]. These published characteristic peptides were used to classify most MSP proteins. The remaining small number of MSP proteins for which a class was still ambiguous were allocated based on their similarities to proteins that had been unambiguously assigned to the three MSP class proteins [24].

## Results

Either spectrum counts or peptide counts can be used as input to relative quantification schemes for proteomic data. Spectrum counts were used in this study because of their demonstrated reproducibility in previous studies [25, 26], and because the Scaffold software automatically deduces normalized spectral values from all samples. Spectrum counts for relative quantification can be controversial because they do not measure physical properties of peptides, and they are based on the assumption that there is linearity of the response between proteins [27]. In this case, because the MSP members are very closely related and therefore should have only a small range in mass, it is likely that the responses were linear enough to meet this assumption.

### Isolation of exosomes

**Fig. 1:**
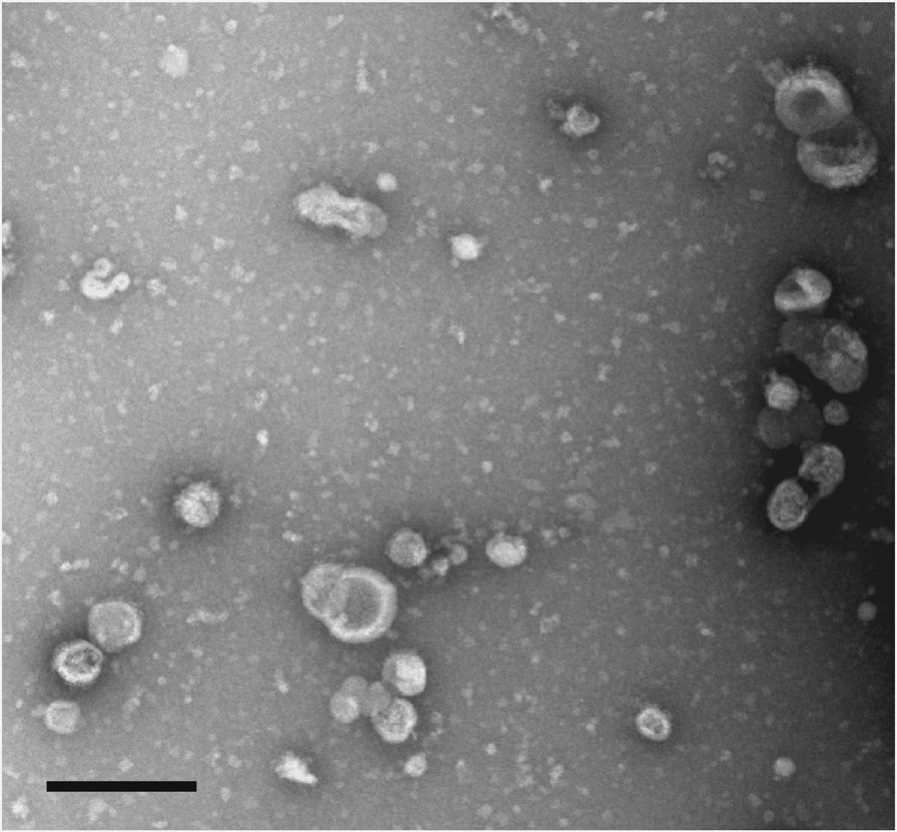
Transmission electron microscopy of the secreted nanovesicles. The secreted nanovesicles from the stationary promastigotes of *L. infantum* were processed and examined by TEM. The size and morphology of the secreted nanovesicles from logarithmic and metacyclic promastigotes were similar (not shown). *Scale-bar*: 200 nm

The secreted nanovesicles under study were released by promastigotes from the same starting cultures, but in different growth phases and conditions. Growth conditions included logarithmic or stationary phase parasites, corresponding to parasites with low or high virulence, respectively, and highly infectious metacyclic promastigote forms isolated from stationary phase cultures. TEM images revealed most of the isolated nanovesicles ranged from 40–60 nm in diameter (Fig. 1), consistent with the size and morphology of exosomes released by other types of cells [28].

### Classes of MSP proteins identified in exosomes released from *L. infantum*

**Fig. 2:**
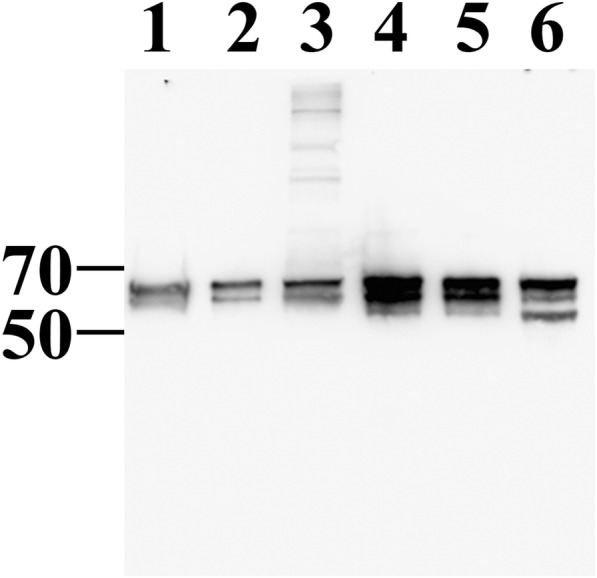
MSP in the secreted nanovesicles of *L. infantum* promastigotes. Seven μg of exosome protein were loaded in each lane. Lanes 1–3: nanovesicles; Lanes 4–6: total cell lysates; Lanes 1 and 4: metacyclic; Lanes 2 and 5: stationary; Lanes 3 and 6: log phase promastigotes. Molecular weight in KDa is shown on the left. The panel was probed with sheep polyclonal antiserum to MPS. One representative among three repeat blots is shown

Three biological replicate exosome preparations each from logarithmic phase, stationary phase, or metacyclic promastigotes were analyzed by LC-MS/MS and examined in the current study. Analysis of the full database, performed previously, used the Distiller function in MASCOT to match total exosome proteins with sequences from the *Leishmania* spp. A western blot of the proteins extracted from secreted nanovesicles and total cell lysates of *L. infantum* from the three development stages, probed with polyclonal antiserum raised against purified *L. infantum* MSP, showed that all nanovesicles contained more than one migrating MSP isoform (Fig. 2). MSP proteins identified in LC-MS/MS mass spectrometric analyses of exosomes were examined further. Seventeen different MSPs were identified, several of which had not yet been identified in *L. infantum* (Table 1).

**Table 1:** MSP proteins identified by LC-MS/MS from exosomes released by *L. infantum* promastigotes

	Protein name	Class	Predicted size (kDa)	Accession number	*Leishmania* spp. with annotation	Mean coverage (%)	No. of peptides involved in class identification
1	LinJ.10.520	S	63	CAM66068	*L. infantum*	37	3
2	LinJ.10.530	S	63.5	CAM66064	*L. infantum*	39	5
3	GP63_LEIDO4	S	64	AAA29237	*L. donovani*	48	5
4	LcMSPL1	L	63.8	P15706	*L. infantum*	40	7
5	GP63_LEIDO	L	63	AAA29244	*L. donovani*	29	4
6	GP63_LEIAM	L	63	Q27673	*L. amazonensis*	8	2
7	GP63_LEIMA	L	64	P08148	*L. major*	12	3
8	LinJ.28.600^a^	C	60.6	CBZ08848	*L. infantum*	21	5
9	LinJ.10.510	C	69	CAM66067	*L. infantum*	30	3
10	LmjF.10.470	C	69	Q4QHH0	*L. major*	16	2
11	LmxM.10.460	C	70	XP_003872882	*L. mexicana*	10	2
12	GP63_LEIME	C	69	P43150	*L. mexicana*	5	1
13	GP63_LEITR	C	70	Q8MNZ1	*L. tropica*	6	1
14	GP63_LEIDO2	C	60	CBY93846	*L. donovani*	9	0
15	GP63_LEIDO3	C	61	CBY93851	*L. donovani*	21	2
16	GP63_LEIDO5	C	39	CAC37955	*L. donovani*	15	2
17	GP63_LEIDO6	C	40	CAC37953	*L. donovani*	28	1

*Note*: The species column indicates the *Leishmania* species in which the protein has been annotated in the database. The mean % coverage is the proportion of amino acids identified per allocated protein and gives an indication of the reliability of the results. Results were restricted to a protein false discovery rate (FDR) of < 0.1% with peptide and protein confidence of 95% and at least two unique peptides required

^a^This was previously renamed as MSP-like protein (MLP) [29]

Each identified MSP protein was assigned a class as described above. MSPS and MSPL class proteins were mainly identified due to the existence of a predicted GPI anchor addition site ‘DGGN’ at AA position 570. Proteins that were predicted to lack a GPI anchor addition site were expected to belong to class C (Additional file 1: Figure S1 and Additional file 2: Figure S2). The additional amino acid similarities between the C classes for all but LinJ.28.600(C) included residues preceding positions 138, 286, 423, 440, 467, 503 and 527 (Additional file 1: Figure S1). Although classified as MSPC here LinJ28.600 was previously renamed as MSP-like protein (MLP) [29].

### Total MSP in exosomes from different parasite developmental stages

Because total promastigote MSP increases as parasites develop from log to stationary phase in vitro, we questioned whether a similar pattern would be observed for MSPs in exosomes. We analyzed first (current section), whether total MSP changes with virulence. Second (below section), we examined whether there were developmental patterns of MSP isoforms in different developmental stages.

### Total MSP in life-cycle stages

**Fig. 3:**
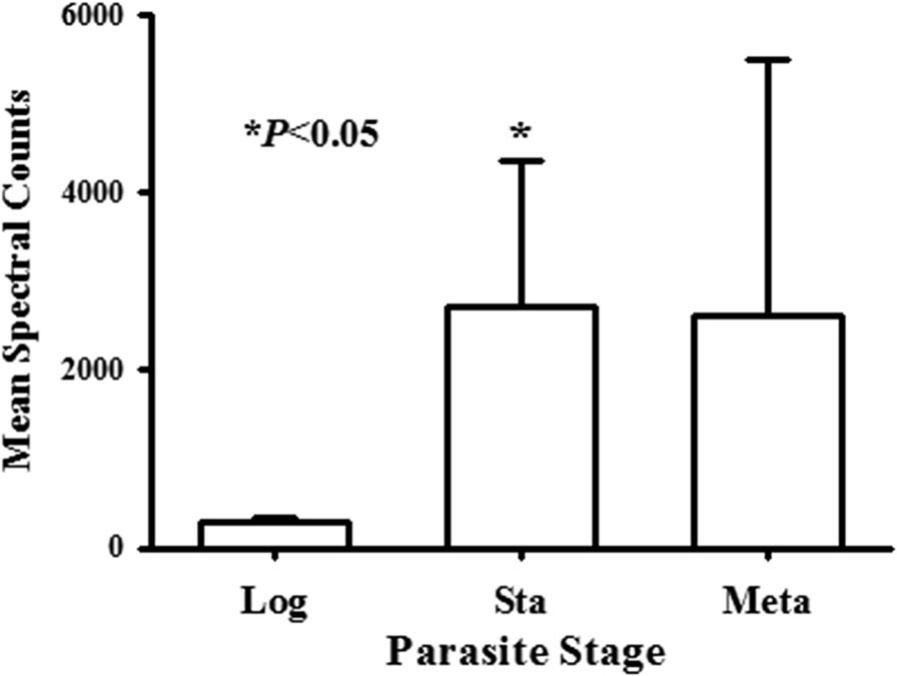
MSP proteins released in exosomes by various stages of promastigotes. The mean ± SE total spectral values per repeat exosome preparation from each life-cycle stage was calculated from sequences of each of the individual exosome samples (*n* = 3). Significant differences were calculated using one-way ANOVA with Tukey *post-hoc* test

To obtain the mean total MSP spectra found in each stage, the individual protein spectral values were added to obtain the total MSP per repeat, and the mean total MSP spectra for the three replicates. Log, stationary phase and metacyclic promastigotes contained mean total spectral values of 304 ± 28, 2702 ± 1658 and 2612 ± 2886, respectively (Fig. 3, Table 2). The difference between total MSP spectra in log versus stationary exosomes was significant (*P* < 0.05) but not between other comparisons (one-way ANOVA, Tukey *post-hoc* test) (Fig. 3). The lack of difference between log and metacyclic exosomes likely was due to the high error between repeats.

**Table 2:** Unique peptide counts of MSP proteins detected in exosomes released from different *L. infantum* promastigote forms. Numbers indicate the number of unique peptides distinguishing each MSP protein in exosomes from each parasite stage (*n* = 3)

MSP	MSP class	Log		Stationary		Metacyclic	
		Unique peptide count	Total peptide quantity	Unique peptide count	Total peptide quantity	Unique peptide count	Total peptide quantity
LinJ.10.520	S	3	114	4	250	5	385
LinJ.10.530	S	20	116	19	261	27	461
GP63_LEIDO4	S	nd	nd	5	342	5	536
LcMSPL1	L	8	139	8	224	7	357
GP63_LEIDO	L	nd	nd	4	393	4	497
GP63_LEIAM	L	nd	nd	2	41	4	50
GP63_LEIMA	L	nd	nd	3	40	3	43
LinJ.28.600	C	4	9	11	52	10	40
LinJ.10.510	C	3	67	3	123	4	190
LmjF.10.470	C	nd	nd	nd	nd	2	79
LmxM.10.460	C	nd	nd	nd	nd	2	47
GP63_LEIME	C	nd	nd	1	16	1	21
GP63_LEITR	C	nd	nd	1	29	1	21
GP63_LEIDO2	C	nd	nd	nd	20	2	30
GP63_LEIDO3	C	nd	nd	2	61	2	110
GP63_LEIDO5	C	nd	nd	2	14	2	44
GP63_LEIDO6	C	nd	nd	1	43	3	50

*Abbreviation*: nd, not detectable

### Differences between individual MSPs or MSPs belonging to different classes

Among the MSPs identified (Table 2), two MSPs [LmjF.10.470(C) and LmxM.10.460(C)] were uniquely present only in isolated metacyclic promastigote-derived exosomes. Thus, MSPS proteins were not uniquely released only by stationary and metacyclic promastigotes, but they were also released in logarithmic phase promastigote exosomes albeit at lower abundance than the others. Therefore, we examined whether the relative abundance of MSP isoforms differed between the different exosome preparations.

**Fig. 4:**
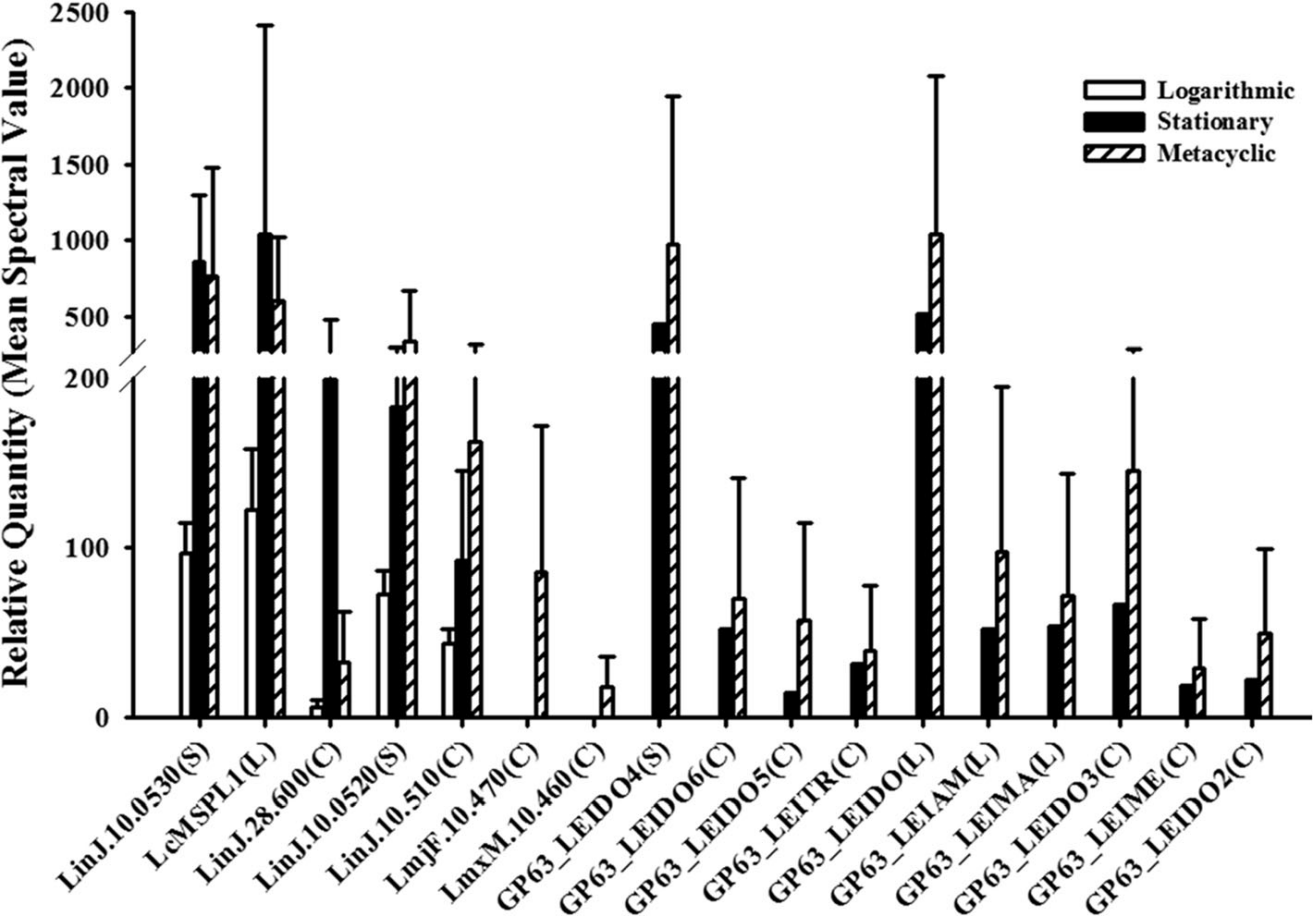
Quantification of individual MSP isoforms in exosomes from log, stationary or metacyclic promastigotes. The relative quantities were calculated according to the spectral values. Data shown as mean ± SD

The relative quantities of each MSP protein were assessed according to normalized numbers of spectra. All MSPs except three followed the pattern of increasing quantity from log to stationary to metacyclic promastigote exosomes. The three exceptions (LinJ.10.530(S), LcMSPL1(L), and LinJ.28.600(C)) increased from log to stationary exosomes but were observed at a level similar or a lower level in exosomes released from metacyclic cells (Fig. 4). Thus, in contrast to the abundance of mRNA [8], MSPL class proteins were not more highly represented in exosomes from logarithmically growing promastigotes than in either stationary or metacyclics. Nonetheless, each protein was differentially represented in exosomes from the different life stages. LinJ.10.530(S), LinJ.10.520(S) and LinJ.10.510(C) were predominantly represented in metacyclic stage exosomes, and only small amounts were present in the log stage exosomes. LcMSPL1(L) and LinJ.28.600(C) were mainly found in stationary phase exosomes, with lower amounts in metacyclic exosomes and relatively low levels in the logarithmic exosomes. The remaining 12 proteins were not detected at all in exosomes from logarithmic stage promastigotes and were represented at their highest level in metacyclic exosomes. There were no MSP isoforms that were present at the highest level in log stage exosomes, even those of the MSPL class.

### Abundance of the MSP classes (MSPC, MSPL or MSPS) in exosomes from the different life forms

**Fig. 5:**
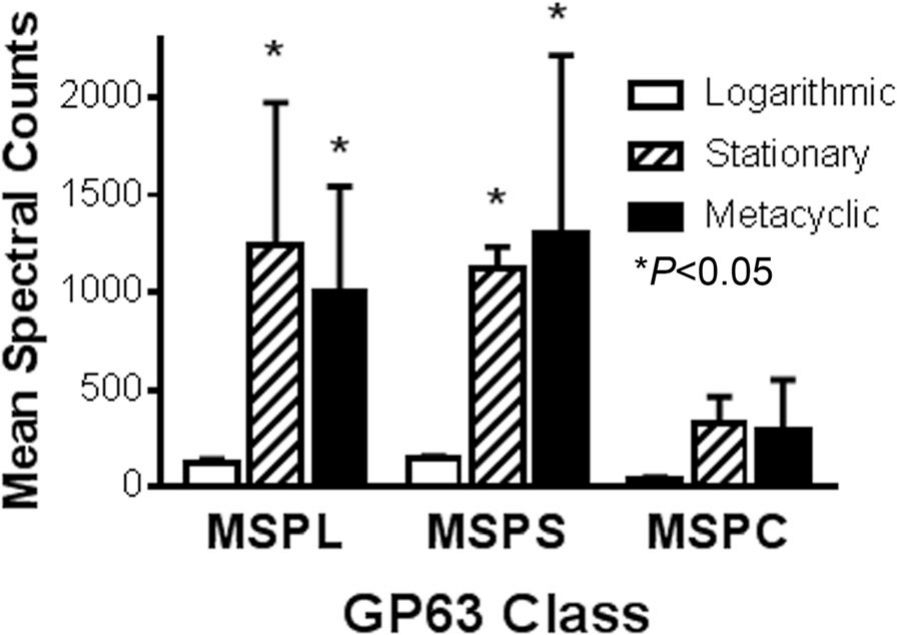
The mean ± SD spectral values corresponding to products of each of the three MSP classes were calculated in exosomes released from logarithmic, stationary or metacyclic promastigotes. MSP classes are MSPL (L), MSPS (S) and MSPC (C). Differences were observed between MSPs in logarithmic exosomes and exosomes from either metacyclic or stationary promastigotes (two-way ANOVA, Tukey *post-hoc* test)

The relative total quantities of proteins derived from the three classes of *msp* genes in exosomes from different stages were calculated according to LC-MS/MS spectra (Fig. 5). There were no significant differences between the abundance of spectra corresponding to MSPC proteins between exosomes from the three different parasite stages. However, the abundance of spectra corresponding to MSPL or to MSPS significantly differed between exosomes from logarithmic parasites and exosomes from either stationary or metacyclic parasites (*P* < 0.05, two-way ANOVA, Tukey *post-hoc* test). The biological significance of these data must be interpreted in light of the absolute quantities of exosomes released by the different life forms. Surprisingly, quantification revealed more exosome protein released from logarithmic compared to either stationary phase or metacyclic promastigotes (17.71, 8.27 and 10.77 μg/10^8^ promastigotes, respectively). This 2.14-fold or 1.65-fold increase in exosomes released by logarithmic compared to stationary, or to metacyclic proamstigotes, respectively, may essentially cancel out any biological consequence of increased abundance released in stationary/metacyclic exosomes.

## Discussion

The *Leishmania* spp. MSPs are highly expressed proteases that contribute in multiple ways to parasite virulence. *msp* genes in a tandem array of at least 18 copies can be categorized as belonging to three classes in *L. infantum*, according to their unique sequence elements in the 3'-UTRs and coding regions [9, 30]. We previously reported differential expression of MSP mRNAs in the different classes. Transcripts of *mspS* genes are detected only in stationary phase and metacyclic promastigotes, *mspL* transcripts are detected only in logarithmic growth, and *mspC* is expressed throughout the growth cycle [8]. We also reported that all three gene classes are constitutively transcribed throughout all growth phases [31], and that differences in *mspL* mRNA abundance are accounted for by differential mRNA stability [32]. Our prior studies of parasite proteins revealed evidence that MSP proteins are differentially represented in *L. infantum* promastigotes from different life-cycle stages [7, 16]. Herein we examined whether the abundance of MSPs is also differentially represented in exosomes released from different developmental forms of *L. infantum* promastigotes. Our analyses revealed multiple MSPs could be identified in exosomes released from all promastigotes, and that logarithmically growing parasites, the least virulent of the types examined, released the lowest amount of total MSP from all three classes. Nonetheless, logarithmic promastigotes released more exosomes per cell than stationary or metacyclic promastigotes, compensating for the lower amount of MSP protein per μg of exosomes. We conclude that the abundance of exosome released must be proportion to the abundance of the different growth stages, respectively.

Direct sequencing of genomic DNA libraries and Southern blotting methods previously showed that there are at least five *mspS*, at least 12 *mspL* and only one *mspC* gene identified in the *L. infantum* genome [8]. However, the annotated *L. infantum* genome (*L. infantum* clone JPCM5, http://tritrypdb.org/tritrypdb/showApplication.do) contains only five annotated *msp* (*gp63*) genes on chromosome 10, likely due to the complexity of aligning tandemly repeated genomic regions with high homology. In addition, two and one *msp* genes are annotated in chromosome 28 and 31, respectively. In the present report we identified seventeen MSP proteins with unique peptides aligning them with either one of the eight *msp* genes in the published *L. infantum* genome, or with reported *msp* (*gp63*) sequences from other species (*L. major*, *L. mexicana*, *L. donovani*, *L. amazonensis* and *L. tropica*). This underscores the fact that our sequence information for the *L. infantum msp* gene cluster is incomplete, using data from both from the original Sanger sequencing/Southern blotting and the more recent high throughput sequencing [16]. Additionally, deep sequencing information will most likely correct this and allow further identification of MSPs and reveal more than the original 3 *msp* gene classes that we highlight herein.

Our analyses of exosome MSP proteins in different gene classes allowed us to identify three or four proteins as belonging to MSPS or MSPL classes, respectively. Ten other proteins were identified as MSPC class members. It is possible that additional information about genomic sequence would lead us to further divide the current MSPC class into products of additional *msp* gene types.

Just as cellular MSP increases in quantity in promastigotes as they develop from log to metacyclic stage [7], our data suggest that MSP also increases in quantity in exosomes released by these parasite forms. Spectra corresponding to total MSP increased approximately 13-fold between exosomes released from log versus metacyclic promastigotes. Prior work indicates that abundant MSP on the surface of promastigotes is advantageous for parasites, enabling them to evade complement lysis and ligate macrophage receptors [33]. Similarly, release of abundant amounts of MSP in exosomes might aid the parasite as it migrates through extracellular matrix after it is introduced through a sand fly bite into host skin [34]. It has been shown in an in vivo study that *Leishmania* promastigotes release exosomes in the midgut of sand flies and exosomes are part of inoculum of sand fly vector along with metacyclic promastigotes. Further, footpad swelling is potentiated by co-inoculation of exosomes isolated from the midgut of sand fly [35].

Our prior published report shows that *mspS* transcripts are expressed most highly during stationary growth when MSP protein is most abundant, and that *mspL* transcripts predominate in logarithmic growth when MSP protein is low [7]. These reports of transcript abundance are consistent with our current observation that MSPSs are more abundant in exosomes from either stationary or metacyclic promastigotes than logarithmic promastigotes. In contrast to mRNA expression patterns, the products of *mspL* genes were not abundantly represented in exosomes from logarithmically growing parasites. The major difference in MSP release was a low amount of all MSP class proteins in exosomes released from logarithmic promastigotes. It seems MSPs of any class are more likely to be packaged into exosomes of stationary or metacyclic promastigotes than less virulent, logarithmically growing promastigotes. Apparently, transcript abundance in the promastigotes is not the main determinant of exosome protein abundance.

Pre-treating monocytes with exosomes has been found to increase *Leishmania* survival, presumably by “priming” the environment to be anti-inflammatory [11]. Studies of other systems have documented uptake of exosomes by host cells [36, 37]. MSPs have been shown to modify macrophage protein tyrosine phosphatases and transcription factors, affecting macrophage activation [12]. MSP has also been shown to interfere with p38_MAPK signaling inside the host cell [38] and may be able to reach the nucleus due to the presence of an NLS-like domain of MSP [39]. From the macrophage nucleus, MSP is able to modify translocation of transcription factors NF-kB and AP-1 [39]. Thus, if exosomes released by *Leishmania* promastigotes are taken up by host macrophages, they could profoundly influence the functional capacity of these macrophages. As such, metacyclic-derived exosomes with high MSP content could have a quite different functional effect than exosomes released by logarithmic parasites.

## Conclusions

*Leishmania* spp. exosomes have been reported to modulate the immune response and lead to increased parasite survival [40]. It is likely that the protein content of exosomes is uniquely suited for host environment modification [40]. MSP (often called GP63 in the literature), a major component of *Leishmania* spp. exosomes, is known to interact with the host environment at several steps of the infectious process. This metalloprotease has been shown to digest extracellular matrix proteins [34], to cleave active complement and generate inactive opsonins [33], and to modify macrophage signaling through cleavage of key transcription factors [41–43]. It seems likely that MSP delivered to the environment by exosomes mediates at least some of these extracellular functions, and ultimately contributes to enhanced parasite survival in different host microenvironments.

## Additional files

Additional file 1: **Figure S1.** Highlight of the peptides and residues identified as indicators of protein class: stationary (S), logarithmic (L), or constitutive (C). The small number after each amino acid section shows the location in the protein sequence. N and C indicate the N-terminal and the C-terminal of the proteins, respectively. See Table 1 for accession numbers. (DOCX 312 kb)

Additional file 2: **Figure S2.** Alignment of all 17 proteins identified. The first four proteins are examples of previously identified S, L, and two C class proteins with accession numbers: LcMSPS1 (Yao 2003) = M80669, LcMSPL1 (Yao et al. [24]) = M80672, LEIDOC1 (Ramamoorthy et al. [8]) = AAA29237.1, MSPC_partial = CAC37969. Accession numbers of the remaining 17 proteins are provided in Table 1. Underlined proteins are identical to already classified proteins: GP63_LEIDO(S) = ‘MSPS4’ in Yao et al. [24]; LcMSPL1(L) = ‘MSPL1’ on NCBI (accession number M80672); GP63_LEIDO6(C) = ‘MSPC’ on NCBI (accession number CAC37953); GP63LEIDO5(C) = ‘constitutive major surface protease’ on NCBI (accession number CAC37955); GP63_LEIME(C) = ‘GP63-C1’ on NCBI (accession number P43150); GP63_LEITR(C) = ‘MSPC’ in Yao et al. [24]. Underlined italics indicate the peptides that were identified by LC-MS/MS. Blue highlight indicates how MSPL1 is differentiated from MSPS1 (Roberts et al. [30]); yellow highlight: suggestive of a C class; black highlight: key features of an S class as described in Roberts et al*.* [9, 30]; grey highlight: areas that show ambiguity towards the classification (due to being a key feature of another class or a non-common feature); light blue highlight: anchor addition site. (DOCX 34 kb)

## Abbreviations

FCS: fetal calf serum
HOMEM: hemoflagellate-modified minimal essential medium
MLP: MSP-like protein
MSP: major surface protease
*mspL*: logarithmic *msp*
*mspS*: stationary *msp*
*mspC*: constitutive *msp*
PBS: phosphate-buffered saline
SFM: serum-free growth medium
SFM: serum-free growth medium
TEM: transmission electron microcope
VL: visceral leishmaniasis

## References

[1] Alvar J, Velez ID, Bern C, Herrero M, Desjeux P, Cano J (2012). Leishmaniasis worldwide and global estimates of its incidence. PLoS One..

[2] Sundar S, Rai M, Chakravarty J, Agarwal D, Agrawal N, Vaillant M (2008). New treatment approach in Indian visceral leishmaniasis: single-dose liposomal amphotericin B followed by short-course oral miltefosine. Clin Infect Dis..

[3] Chappuis F, Sundar S, Hailu A, Ghalib H, Rijal S, Peeling RW (2007). Visceral leishmaniasis: what are the needs for diagnosis, treatment and control?. Nat Rev Microbiol..

[4] Rosenzweig D, Smith D, Opperdoes F, Stern S, Olafson RW, Zilberstein D (2008). Retooling *Leishmania* metabolism: from sand fly gut to human macrophage. FASEB J..

[5] Yao C (2010). Major surface protease of trypanosomatids: one size fits all?. Infect Immun..

[6] Besteiro S, Williams RA, Coombs GH, Mottram JC (2007). Protein turnover and differentiation in *Leishmania*. Int J Parasitol..

[7] Yao C, Leidal KG, Brittingham A, Tarr DE, Donelson JE, Wilson ME (2002). Biosynthesis of the major surface protease GP63 of *Leishmania chagasi*. Mol Biochem Parasitol..

[8] Ramamoorthy R, Donelson JE, Paetz KE, Maybodi M, Roberts SC, Wilson ME (1992). Three distinct RNAs for the surface protease gp63 are differentially expressed during development of *Leishmania donovani chagasi* promastigotes to an infectious form. J Biol Chem..

[9] Roberts SC, Swihart KG, Agey MW, Ramamoorthy R, Wilson ME, Donelson JE (1993). Sequence diversity and organization of the msp gene family encoding gp63 of *Leishmania chagasi*. Mol Biochem Parasitol..

[10] Yao C, Donelson JE, Wilson ME (2007). Internal and surface-localized major surface proteases of *Leishmania* spp. and their differential release from promastigotes. Eukaryot Cell..

[11] Silverman JM, Clos J, Horakova E, Wang AY, Wiesgigl M, Kelly I (2010). *Leishmania* exosomes modulate innate and adaptive immune responses through effects on monocytes and dendritic cells. J Immunol..

[12] Hassani K, Shio MT, Martel C, Faubert D, Olivier M (2014). Absence of metalloprotease GP63 alters the protein content of *Leishmania* exosomes. PLoS One..

[13] Santarem N, Racine G, Silvestre R, Cordeiro-da-Silva A, Ouellette M (2013). Exoproteome dynamics in *Leishmania infantum*. J Proteomics..

[14] Silverman JM, Clos J, de'Oliveira CC, Shirvani O, Fang Y, Wang C (2010). An exosome-based secretion pathway is responsible for protein export from *Leishmania* and communication with macrophages. J Cell Sci..

[15] Berens RL, Brun R, Krassner SM (1976). A simple monophasic medium for axenic culture of hemoflagellates. J Parasitol..

[16] Yao C, Luo J, Storlie P, Donelson JE, Wilson ME (2004). Multiple products of the *Leishmania chagasi* major surface protease (MSP or GP63) gene family. Mol Biochem Parasitol..

[17] Yao C, Chen Y, Sudan B, Donelson JE, Wilson ME (2008). *Leishmania chagasi*: homogenous metacyclic promastigotes isolated by buoyant density are highly virulent in a mouse model. Exp Parasitol..

[18] Raposo G, Nijman HW, Stoorvogel W, Liejendekker R, Harding CV, Melief CJ (1996). B lymphocytes secrete antigen-presenting vesicles. J Exp Med..

[19] Wilson ME, Hardin KK, Donelson JE (1989). Expression of the major surface glycoprotein of *Leishmania donovani chagasi* in virulent and attenuated promastigotes. J Immunol..

[20] Shevchenko A, Tomas H, Havlis J, Olsen JV, Mann M (2006). In-gel digestion for mass spectrometric characterization of proteins and proteomes. Nat Protoc..

[21] Rappsilber J, Mann M, Ishihama Y (2007). Protocol for micro-purification, enrichment, pre-fractionation and storage of peptides for proteomics using StageTips. Nat Protoc..

[22] Perkins DN, Pappin DJ, Creasy DM, Cottrell JS (1999). Probability-based protein identification by searching sequence databases using mass spectrometry data. Electrophoresis..

[23] Larkin MA, Blackshields G, Brown NP, Chenna R, McGettigan PA, McWilliam H (2007). Clustal W and Clustal X version 2.0. Bioinformatics..

[24] Yao C, Donelson JE, Wilson ME (2003). The major surface protease (MSP or GP63) of *Leishmania* sp. Biosynthesis, regulation of expression, and function. Mol Biochem Parasitol..

[25] Liu H, Sadygov RG, Yates JR (2004). A model for random sampling and estimation of relative protein abundance in shotgun proteomics. Anal Chem..

[26] Zhang B, VerBerkmoes NC, Langston MA, Uberbacher E, Hettich RL, Samatova NF (2006). Detecting differential and correlated protein expression in label-free shotgun proteomics. J Proteome Res..

[27] Bantscheff M, Lemeer S, Savitski MM, Kuster B (2012). Quantitative mass spectrometry in proteomics: critical review update from 2007 to the present. Anal Bioanal Chem..

[28] Mathivanan S, Ji H, Simpson RJ (2010). Exosomes: extracellular organelles important in intercellular communication. J Proteomics..

[29] Yao C, Gaur Dixit U, Barker JH, Teesch LM, Love-Homan L, Donelson JE (2013). Attenuation of *Leishmania infantum* chagasi metacyclic promastigotes by sterol depletion. Infect Immun..

[30] Roberts SC, Wilson ME, Donelson JE (1995). Developmentally regulated expression of a novel 59-kDa product of the major surface protease (Msp or gp63) gene family of *Leishmania chagasi*. J Biol Chem..

[31] Wilson ME, Paetz KE, Ramamoorthy R, Donelson JE (1993). The effect of ongoing protein synthesis on the steady state levels of Gp63 RNAs in *Leishmania chagasi*. J Biol Chem..

[32] Brittingham A, Miller MA, Donelson JE, Wilson ME (2001). Regulation of GP63 mRNA stability in promastigotes of virulent and attenuated *Leishmania chagasi*. Mol Biochem Parasitol..

[33] Brittingham A, Morrison CJ, McMaster WR, McGwire BS, Chang KP, Mosser DM (1995). Role of the *Leishmania* surface protease gp63 in complement fixation, cell adhesion, and resistance to complement-mediated lysis. J Immunol..

[34] McGwire BS, Chang KP, Engman DM (2003). Migration through the extracellular matrix by the parasitic protozoan *Leishmania* is enhanced by surface metalloprotease gp63. Infect Immun..

[35] Atayde VD, Aslan H, Townsend S, Hassani K, Kamhawi S, Olivier M (2015). Exosome secretion by the parasitic protozoan *Leishmania* within the sand fly midgut. Cell Rep..

[36] Marcilla A, Trelis M, Cortés A, Sotillo J, Cantalapiedra F, Minguez MT (2012). Extracellular vesicles from parasitic helminths contain specific excretory/secretory proteins and are internalized in intestinal host cells. PLoS One..

[37] Abels ER, Breakefield XO (2016). Introduction to extracellular vesicles: biogenesis, RNA cargo selection, content, release, and uptake. Cell Mol Neurobiol..

[38] Halle M, Gomez MA, Stuible M, Shimizu H, McMaster WR, Olivier M (2009). The *Leishmania* surface protease GP63 cleaves multiple intracellular proteins and actively participates in p38 mitogen-activated protein kinase inactivation. J Biol Chem..

[39] Isnard A, Christian JG, Kodiha M, Stochaj U, McMaster WR, Olivier M (2015). Impact of *Leishmania* infection on host macrophage nuclear physiology and nucleopore complex integrity. PLoS Pathog..

[40] Silverman JM, Reiner NE (2011). *Leishmania* exosomes deliver preemptive strikes to create an environment permissive for early infection. Front Cell Infect Microbiol..

[41] Contreras I, Gomez MA, Nguyen O, Shio MT, McMaster RW, Olivier M (2010). *Leishmania*-induced inactivation of the macrophage transcription factor AP-1 is mediated by the parasite metalloprotease GP63. PLoS Pathog..

[42] Gomez MA, Contreras I, Halle M, Tremblay ML, McMaster RW, Olivier M (2009). *Leishmania* GP63 alters host signaling through cleavage-activated protein tyrosine phosphatases. Sci Signal..

[43] Isnard A, Shio MT, Olivier M (2012). Impact of *Leishmania* metalloprotease GP63 on macrophage signaling. Front Cell Infect Microbiol..

