# Supplementary material for: Extracellular release of virulence factor major surface protease via exosomes in Leishmania infantum promastigotes
Source: Parasit Vectors. 2018 Jun 19;11:355. doi: 10.1186/s13071-018-2937-y (PMC6006689; doi:10.1186/s13071-018-2937-y)
Supplement: Supplementary file 1 — Figure S1. Highlight of the peptides and residues identified as indicators of protein class: stationary (S), logarithmic (L), or constitutive (C). The small number after each amino acid section shows the location in the protein sequence. N and C indicate the N-terminal and the C-terminal of the proteins, respectively. See Table 1 for accession numbers. (DOCX 312 kb) [file 13071_2018_2937_MOESM1_ESM.docx]

**Additional file 1: Figure S1.** Highlight of the peptides and residues identified as indicators of protein class; Stationary (S), Logarithmic (L), or Constitutive (C). The small number after each amino acid section shows the location in the protein sequence. N and C indicate the N-terminal and the C-terminal of the proteins, respectively. See Table 1 for accession numbers.


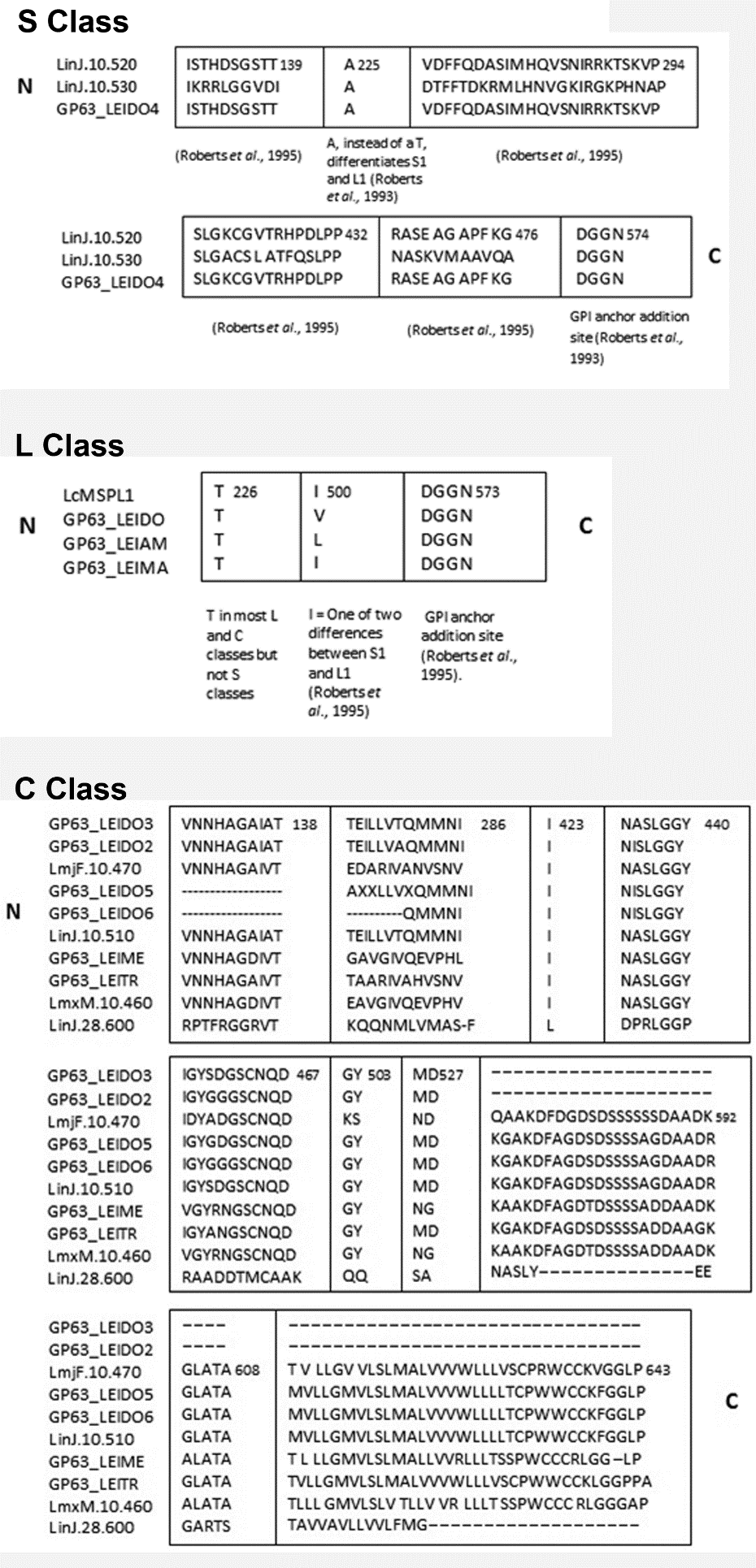


[9]. Roberts SC, Swihart KG, Agey MW, Ramamoorthy R, Wilson ME, Donelson JE. Sequence diversity and organization of the msp gene family encoding gp63 of *Leishmania chagasi*. Mol Biochem Parasitol. 1993;62:157-71.

[30]. Roberts SC, Wilson ME, Donelson JE. Developmentally regulated expression of a novel 59-kDa product of the major surface protease (Msp or gp63) gene family of *Leishmania chagasi*. J Biol Chem. 1995;270:8884-92.
