# Supplementary material for: Extracellular release of virulence factor major surface protease via exosomes in Leishmania infantum promastigotes
Source: Parasit Vectors. 2018 Jun 19;11:355. doi: 10.1186/s13071-018-2937-y (PMC6006689; doi:10.1186/s13071-018-2937-y)
Supplement: Supplementary file 2 — Figure S2. Alignment of all 17 proteins identified. The first four proteins are examples of previously identified S, L, and two C class proteins with accession numbers: LcMSPS1 (Yao 2003) = M80669, LcMSPL1 (Yao et al. [24]) = M80672, LEIDOC1 (Ramamoorthy et al. [8]) = AAA29237.1, MSPC_partial = CAC37969. Accession numbers of the remaining 17 proteins are provided in Table 1. Underlined proteins are identical to already classified proteins: GP63_LEIDO(S) = ‘MSPS4’ in Yao et al. [24]; LcMSPL1(L) = ‘MSPL1’ on NCBI (accession number M80672); GP63_LEIDO6(C) = ‘MSPC’ on NCBI (accession number CAC37953); GP63LEIDO5(C) = ‘constitutive major surface protease’ on NCBI (accession number CAC37955); GP63_LEIME(C) = ‘GP63-C1’ on NCBI (accession number P43150); GP63_LEITR(C) = ‘MSPC’ in Yao et al. [24]. Underlined italics indicate the peptides that were identified by LC-MS/MS. Blue highlight indicates how MSPL1 is differentiated from MSPS1 (Roberts et al. [30]); yellow highlight: suggestive of a C class; black highlight: key features of an S class as described in Roberts et al. [9, 30]; grey highlight: areas that show ambiguity towards the classification (due to being a key feature of another class or a non-common feature); light blue highlight: anchor addition site. (DOCX 34 kb) [file 13071_2018_2937_MOESM2_ESM.docx]

**Additional file 2: Figure S2.** Alignment of all seventeen proteins identified. The first four proteins are examples of previously identified S, L, and two C class proteins with accession numbers: LcMSPS1 (Yao 2003) = M80669, LcMSPL1 (Yao *et al*., [24]) = M80672, LEIDOC1 (Ramamoorthy *et al*., [8]) = AAA29237.1, MSPC_partial = CAC37969. Accession numbers of the seventeen remaining proteins can be seen in Table 1. Underlined proteins are identical to already classified proteins: GP63_LEIDO(S) = ‘MSPS4’ in Yao *et al*., [24]; LcMSPL1(L) = ‘MSPL1’ on NCBI (accession number M80672); GP63_LEIDO6(C) = ‘MSPC’ on NCBI (accession number CAC37953); GP63LEIDO5(C) = ‘constitutive major surface protease’ on NCBI (accession number CAC37955); GP63_LEIME(C) = ‘GP63-C1’ on NCBI (accession number P43150); GP63_LEITR(C) = ‘MSPC’ in Yao *et al*., [24]. Underlined italics indicate the peptides that were identified by LC-MS/MS. Blue highlight = how MSPL1 is differentiated from MSPS1 (Roberts *et al*., [30]). Yellow highlight = suggestive of a C class. Black highlight = key features of an S class as described in Roberts *et al.,* [30, 9]. Grey highlight = areas that show ambiguity towards the classification (due to being a key feature of another class or a non-common feature). Light blue highlight = anchor addition site.

LcMSPS1 --------------------------------------------MSVDSSSTHRHRSVAA 16

LcMSPL1 --------------------------------------------MSVDSSSTHRHRSVAA 16

LEIDOC1 --------------------------------------------MSVDSSSTHRHRSVAA 16

MSPC_partial ------------------------------------------------------------

LinJ.10.0520(S) --------------------------------------------MSVDSSSTHRHRSVAA 16

LinJ.10.0530(S) --------------------------------------------MSVDSSSTHRHRSVAA 16

GP63_LEIDO4(S) --------------------------------------------MSVDSSSTHRHRSVAA 16

LcMSPL1(L) --------------------------------------------MSVDSSSTHRHRSVAA 16

GP63_LEIDO(L) --------------------------------------------MSVDSSSTHRHRSVAA 16

GP63_LEIAM(L) --------------------------------------------MSVDSSSTHRHR*CVAA* 16

GP63_LEIMA(L) --------------------------------------------MSVDSSSTHRRRCVAA 16

GP63_LEIDO3(C) --------------------------------------------MSVDSSSTHRHRSVAA 16

GP63_LEIDO2(C) --------------------------------------------MSVDSSSTHRHRSVAA 16

LmjF.10.470(C) --------------------------------------------MSVDSSSTHRRRCVAA 16

GP63_LEIDO6(C) ------------------------------------------------------------

GP63_LEIDO5(C) ------------------------------------------------------------

LinJ.10.510(C) --------------------------------------------MSVDSSSTHRHRSVAA 16

GP63_LEIME(C) --------------------------------------------MPVDSSSTHRHRCVAA 16

GP63_LEITR(C) ------------------------------------------MSVDSSSSSTHRRRCVAA 18

LmxM.10.460(C) --------------------------------------------MPVDSSSTHRHRCVAA 16

LinJ.28.600(C?) ---------------------------------------------------------MRR 3

LcMSPS1 RLVRLAAAGAAVIAAVGTAAAWAHAG---AVQHRCIHDAMQARVRQSVARHHTAPGAVSA 73

LcMSPL1 RLVRLAAAGAAVIAAVGTAAAWAHAG---AVQHRCIHDAMQARVRQSVARHHTAPGAVSA 73

LEIDOC1 RLVRLAAAGAAVIAAVGTAAAWAHAG---AVQHRCIHDAMQARVRQSVARHHTAPGAVSA 73

MSPC_partial ------------------------------------------------------------

LinJ.10.0520(S) RLVRLAAAGAAVIAAVGTAAAWAHAG---AVQHRCIHDAMQARVRQSVARHHTAPGAVSA 73

LinJ.10.0530(S) RLVRLAAAGAAVIAAVGTAAAWAHAG---AVQHRCIHDAMQARVRQSVARHHTAPGAVSA 73

GP63_LEIDO4(S) RLVRLAAAGAAVIAAVGTAAAWAHAG---AVQHRCIHDAMQARVRQSVARHHTAPGAVSA 73

LcMSPL1(L) RLVRLAAAGAAVIAAVGTAAAWAHAG---AVQHRCIHDAMQARVRQSVARHHTAPGAVSA 73

GP63_LEIDO(L) RLVRLAAAGAAVIAAVGTAAAWAHAG---AVQHRCIHDAMQARVRQSVARHHTAPGAVSA 73

GP63_LEIAM(L) *RLVPLAAAGAAVTVAVGTAAAW*AHAG---AVQHRCIHDAMQARVRQSVAAQRMAPSAVSA 73

GP63_LEIMA(L) RLVRLAAAGAAVTVAVGTAAAWAHAG---ALQHRCVHDAMQARVRQSVADHHKAPGAVSA 73

GP63_LEIDO3(C) RLVRLAAAGAAVIAAVGTAAAWAHAG---AVQHRCIHDAMQARVRQSVARHHTAPGAVSA 73

GP63_LEIDO2(C) RLVRLAAAGAAVIAAVGTAAAWAHAG---AVQHRCIHDAMQPRVRQSVARHHTAPGAVSA 73

LmjF.10.470(C) RLVRLAAAGAAVTVAVGTAAAWAHAG---ALQHRCVHDAMQARVRQSVADHHKAPGAVSA 73

GP63_LEIDO6(C) ------------------------------------------------------------

GP63_LEIDO5(C) ------------------------------------------------------------

LinJ.10.510(C) RLVRLAAAGAAVIAAVGTAAAWAHAG---AVQHRCIHDAMQARVRQSVARHHTAPGAVSA 73

GP63_LEIME(C) PLVRLAAAGAAVTVAVGTAAAWAHAG---APQHRCIHDAMQARVLQSVAAQRMAPSAVSA 73

GP63_LEITR(C) RLVRLAAAGAAVTVAVGTAAAWAHAG---ALQHRCIHDAMQARVRQSVARHHTAPGAVSA 75

LmxM.10.460(C) RLVRLAAAGAAVTVAVGTAAAWAHAG---APQHRCIHDAMQARVLQSVAAQRMAPSAVSA 73

LinJ.28.600(C?) TLLGIAVAFALVCCVVGAGAAQGDPERADSEEPRCGFDELEARMIG------TRVSVISR 57

LcMSPS1 VGLPYVTLD---TAAAADR--RPGSAPTVVRAANWGALRIAVSTEDLTDPAYHCARVGQH 128

LcMSPL1 VGLPYVTLD---TAAAADR--RPGSAPTVVRAANWGALRIAVSTEDLTDPAYHCARVGQH 128

LEIDOC1 VGLPYVTLD---TAAAADR--RPGSAPTVVRAANWGALRIAVSTEDLTDPAYHCARVGQR 128

MSPC_partial ------------------------------------------------------------

LinJ.10.0520(S) VGLPYVTLD---TAAAADR--*RPGSAPTVVRAANWGALRIAVSTEDLTDPAYHCAR*VGQR 128

LinJ.10.0530(S) VGLPYVTLD---TAAAADR--*RPGSAPTVVRAANWGALRIAVSTEDLTDPAYHCAR*VGQH 128

GP63_LEIDO4(S) VGLPYVTLD---TAAAADR--RPGSAPTVVRA*ANWGALRIAVSTEDLTDPAYHCAR*VGQR 128

LcMSPL1(L) VGLPYVTLD---TAAAADR--*RPGSAPTVVRAANWGALRIAVSTEDLTDPAYHCAR*VGQH 128

GP63_LEIDO(L) VGLSYVTL---------------GAAPTVVR*AANWGALRIAVSTEDLTD*SAYHCARVGQR 118

GP63_LEIAM(L) VGLPHVTLDAGNTAAGADP--STGTAN-VVR*AANWGALR*IAVSAEDLTDPAYHCARVGQR 130

GP63_LEIMA(L) VGLPYVTLDAAHTAAAADP--RPGSARSVVRDVNWGALR*IAVSTEDLTDPAYHCAR*VGQH 131

GP63_LEIDO3(C) VGLPYVTLD---TAAAADR--RPGSAPTVVR*AANWGALRIAVSTEDLTDPAYHCAR*VGQR 128

GP63_LEIDO2(C) VGLPYVTLD---TAAAADR--RPGSAPTVVR*AANWGALR*IAVSTEDLTDSAYHCARVGQR 128

LmjF.10.470(C) VGLPYVTLDAAHTAAAADP--RPGSARSVVRDVNWGALR*IAVSTEDLTDPAYHCAR*VGQR 131

GP63_LEIDO6(C) ------------------------------------------------------------

GP63_LEIDO5(C) ------------------------------------------------------------

LinJ.10.510(C) VGLPYVTLD---TAAAADR--*RPGSAPTVVRAANWGALRIAVSTEDLTDPAYHCAR*VGQR 128

GP63_LEIME(C) VGLPYVSVVPVENASTLDYSLSDSTSPGVVR*AANWGALR*VAVSAEDLTDPAYHCARVGQQ 133

GP63_LEITR(C) VGLPYVTLDAAHTAAAADP--RPGSAPTVVRAANWSTLRVAVSTEDLTDPAYHCARVGQR 133

LmxM.10.460(C) VGLPYVSVVPVENASTLDYSLSDSTSPGVVR*AANWGALR*IAVSAEDLTDPAYHCARVGQR 133

LinJ.28.600(C?) VEPP-----------------TGELAVAA*AATGAWQPIR*IAVFTEDISNSSQHCTASGQS 100

LcMSPS1 IKRRLGGVDICTAEDILTDEKRDILVKHLIPQALQLHTERLKVRQVQDKWKVTGMG--DD 186

LcMSPL1 IKRRLGGVDICTAEDILTDEKRDILVKHLIPQALQLHTERLKVRQVQDKWKVTGMG--DD 186

LEIDOC1 VNNHAGAIATCTAEDILTDEKRDILVKYLIPQALQLHTERLKVRQVQDKWNVTGMV--DE 186

MSPC_partial ------------------------------------------------------------

LinJ.10.0520(S) *ISTHDSGSTTCTAEDILTDEKR*DILVK*HLIPQALQLHTER*LKVRQVQDKWK*VTGMD*--*DD* 186

LinJ.10.0530(S) IKRR*LGGVDICTAEDILTDEKRDILVKHLIPQALQLHTER*LKVRQVQDKWK*VTGMG*--*DD* 186

GP63_LEIDO4(S) *ISTHDSGSTTCTAEDILTDEKR*DILVK*HLIPQALQLHTER*LKVRQVQDKWK*VTGMD--DD* 186

LcMSPL1(L) IKR*RLGGVDICTAEDILTDEKRDILVKHLIPQALQLHTER*LKVRQVQDKWK*VTGMG*--*DD* 186

GP63_LEIDO(L) ISTRDGRFAI*CTAEDILTDEKR*DILVKYLIPQALQLHTERLKVRQVQDKWKVTGMG--NE 176

GP63_LEIAM(L) VNNHVGDIVT*CTAEDILTDEKR*DILVKHLVPQALQLHRERLKVQQVQGKWKVTGMT--AD 188

GP63_LEIMA(L) VKDHAGAIVTCTAEDILTNEKRDILVKHLIPQAVQLHTERLKVQQVQGKWKVTDMV--GD 189

GP63_LEIDO3(C) VNNHAGAIATCTAEDILTDEKRDILVKYLIPQALQLHTERLKVRQVQDKWNVTDMV--DE 186

GP63_LEIDO2(C) VNNHAGAIATCTAEDILSDEKRDILVKYLIPQALQLHTERLKVRQVQDKWKVTDMV--DE 186

LmjF.10.470(C) VNNHAGAIVTCTAEDILTNEKRNILVKYLIPQAVQLHKERLKVQQVQGKWKVTDMV--GE 189

GP63_LEIDO6(C) ------------------------------------------------------------

GP63_LEIDO5(C) ------------------------------------------------------------

LinJ.10.510(C) *VNNHAGAIATCTAEDILTDEKR*DILVK*YLIPQALQLHTER*LKVRQVQDKWNVTGMV--DE 186

GP63_LEIME(C) VNNHAGDIVT*CTAEDILTDEKR*DTLVKHLVPQALQLHRERLKVRQVQGKWKVTGMA--DV 191

GP63_LEITR(C) *VNNHAGAIVTCTAEDILTDEKR*DILRKYLIPQALQLHTERLKARQVQGKWKVTGMV--DE 191

LmxM.10.460(C) VNNH*AGDIVTCTAEDILTDEKR*DTLVKHLVPQALQLHRERLKVRQVQGKWKVTGMA--AD 191

LinJ.28.600(C?) RPTFRGGR*VTCSAADVLTR*EKKR*VLLELLIPSAVQLHQER*LNVQR*ENGNIVVSPFIK*K*NS* 160

LcMSPS1 VCSDFKVPPAHITDGLSNTDFVMYVASVPSEEGVLAWAATCQVFSDGHPAVGVINIPAAN 246

LcMSPL1 VCSDFKVPPAHITDGLSNTDFVMYVASVPSEEGVLAWATTCQVFSDGHPAVGVINIPAAN 246

LEIDOC1 ICGDFKVPPAHITEGFSNTDFVMYVASVPSEEGVLAWATTCQVFSDGHPAVGVINIPAAN 246

MSPC_partial ------------------------------------------------------------

LinJ.10.0520(S) *VCSDFK*V*PPAHITDGLSNTDFVMYVASVPSEEGVLAWAATCQVFSDGHPAVGVVNIPAAN* 246

LinJ.10.0530(S) *VCSDFKVPPAHITDGLSNTDFVMYVASVPSEGDVLAWAATCQVFSDGHPAVGVINIPAAN* 246

GP63_LEIDO4(S) *VCSDFK*VPPAHITDGLSNTDFVMYVASVPSEEGVLAWAATCQVFSDGHPAVGVVNIPAAN 246

LcMSPL1(L) *VCSDFKVPPAHITDGLSNTDFVMY*VASVPSEEGVLAWATTCQVFSD*GHPAVGVINIPAAN* 246

GP63_LEIDO(L) ICGHFKVPPAHITDGLSNTDFVMYVASVPSEGDVLAWATTCQVFSDGHPAVGVINIPAAN 236

GP63_LEIAM(L) VCRYFKVPPAHVTGGVTNTDFVLYVASVPSEESVLAWATTCQVFADGHPAVGVINIPAAN 248

GP63_LEIMA(L) ICGDFKVPQAHITEGFSNTDFVMYVASVPSEEGVLAWATTCQTFSDGHPAVGVINIPAAN 249

GP63_LEIDO3(C) ICGDFKVPQAHITEGFSNTDFVMYVASVPSEEGVLAWATTCQVFSDGHPAVGVINIPAAN 246

GP63_LEIDO2(C) ICGDFKVPPAHITDGLSNTDFVMYVASVPSEEDVXAWATTCQVFSDGHPAVGVINIPAAN 246

LmjF.10.470(C) ICGDFKVPQAHITEGFSNTDFVMYVASVPSEEGVLAWATTCQTFSDGHPAVGVINIPAAN 249

GP63_LEIDO6(C) ------------------------------------------------------------

GP63_LEIDO5(C) ------------------------------------------------------------

LinJ.10.510(C) ICGDFKVPPAHITEGFSNTDFVMYVASVPSEEGVLAWATTCQVFSD*GHPAVGVINIPAAN* 246

GP63_LEIME(C) ICGDFKVPPEHITEGVTNTDFVLYVASVPSEESVLAWATTCQVFPDGHPAVGVINIPAAN 251

GP63_LEITR(C) ICGDFKVPQAHITEGFSNTDFVMYVASVPSEEGVLAWATTCQVFSDGHPAVGVINIPAAN 251

LmxM.10.460(C) VCGYFKVPPEHITEGVTNTDFVLYVASVPSEESVLAWATTCQVFAD*GHPAVGVINIPAAN* 251

LinJ.28.600(C?) *ICGQFSIPEEHMKTGVPDADFVLYMSAAPTSGSVIAWAVKCQSFDNGRPSVGVATISPK*Y 220

LcMSPS1 IASRYDQLVTRVVTHEMAHALGFSVGFFEGARILESISNVRHKDFDVPVINSSTAVAKAR 306

LcMSPL1 IASRYDQLVTRVVTHEMAHALGFSVGFFEGARILESISNVRHKDFDVPVINSSTAVAKAR 306

LEIDOC1 IASRYDQLVTRVVTHEMAHVG-FSGTFFTEILLVTQMMNIRGKDFNVSVINSSTAVAKAR 305

MSPC_partial -------------------------NVLTEILLVTQMMNIRGKDFNVSVINSSTAVAKAR 35

LinJ.10.0520(S) *IASRYNQLVTR*VVTHEMAHTLGFSVDFFQDASIMHQVSNIRRK*TSKVPVLK*SRTAVAK*AR* 306

LinJ.10.0530(S) *IASRYDQLVTRVVTHEMAHALGFSDTFFTDKRMLHNVGK*IRGKPHNAPVINSSTAVAK*AR* 306

GP63_LEIDO4(S) IASR*YNQLVTR*VVTHEMAHTLGFSVDFFQDASIMHQVSNIRRKTSKVPVLKSRTAVAKAR 306

LcMSPL1(L) *IASRYDQLVTRVVTHEMAHALGFSVGFFEGARILESISNVR*HKDFDVPVINSSTAVAK*AR* 306

GP63_LEIDO(L) IASR*YDQLVTR*VVTHEMAHALGFSVVFFRDAR*ILESISNVR*HKDFDVPVINSSTAVAKAR 296

GP63_LEIAM(L) IASR*YDQLVT*RVVAHEMAHALGFSGTFFDRVGIVQKVPDVRGKPYFTPMINSSTAVAKAR 308

GP63_LEIMA(L) IASR*YDQLVTR*VVTHEMAHALGFSGPFFEDARIVANVPNVRGKNFDVPVINSSTAVAKAR 309

GP63_LEIDO3(C) IASR*YDQLVTR*VVTHEMAHALGFSGTFFTEILLVTQMMNIRGKDFDVPVINSSTAVAKAR 306

GP63_LEIDO2(C) IASR*YDQLVTR*VVTHEMAHALGFSGTFFTEILLVAQMMNIRGKDFNVPVINSSTAVAKAR 306

LmjF.10.470(C) IASR*YDQLVTR*VVTHEMAHALGFSGPFFEDARIVANVSNVRGKNFDVPVINSSTAVAKAR 309

GP63_LEIDO6(C) --------------------------XFAXXLLVXQMMNIRGKDFNVSVINSSTAVAKAR 34

GP63_LEIDO5(C) -----------------------------------QMMNIRGKDFNVPVINSSTAVAKAR 25

LinJ.10.510(C) *IASRYDQLVTR*VVTHEMAHALGFSGTFFTEILLVTQMMNIRGKDFNVSVINSSTAVAK*AR* 306

GP63_LEIME(C) IASR*YDQLVTR*VVTHEMAHAVGFSGTFFGAVGIVQEVPHLRRKDFNVSVITSSTVVAKAR 311

GP63_LEITR(C) IASR*YDQLVTR*VVTHEMAHALGFSEEFFTAARIVAHVSNVRHKTLKVPVVNSSTAV-KAR 310

LmxM.10.460(C) *IASRYDQLVTR*VVTHEMAHALGFSDTFFEAVGIVQEVPHVRGKDFNVSVITSSTAVAKAR 311

LinJ.28.600(C?) IT--AEPK*TVRVVAHEVLHALGFTR*SVFKQQNMLVMAS-FRGK-SPSPVIRSANVVAQAQ 276

.* * .:: * ..* :*:

LcMSPS1 EQYGCDTLEYLEIEDQGGAGSAGSHIKMRNAQDELMAPAAAAGYYSALTMAIFQDLGFYQ 366

LcMSPL1 EQYGCDTLEYLEIEDQGGAGSAGSHIKMRNAQDELMAPAAAAGYYSALTMAIFQDLGFYQ 366

LEIDOC1 EQYGCDTLEYLEIEDQGGAGSAGSHIKMRNAKDELMAPAAAAGYYSALTMAIFQDLGFYQ 365

MSPC_partial EQYGCDTLEYLEIEDQGGAGSAGSHIKMRNAKDELMAPAAAAGYYSALTMAIFQDLGFYQ 95

LinJ.10.0520(S) *EQYGCDTLEYLEIEDQGGAGSAGSHIK*MR*NAQDELMAPAAAAGYYSALTMAIFQDLGFYQ* 366

LinJ.10.0530(S) *EQYGCDTLEYLEMEDEGGAVSAGSHIK*MR*NAQDELMAPAAAAGYYSALTMAIFQDLGFYQ* 366

GP63_LEIDO4(S) *EQYGCDTLEYLEIEDQGGAGSAGSHIK*MR*NAQDELMAPAAAAGYYSALTMAIFQDLGFYQ* 366

LcMSPL1(L) *EQYGCDTLEYLEIEDQGGAGSAGSHIK*MR*NAQDELMAPAAAAGYYSALTMAIFQDLGFYQ* 366

GP63_LEIDO(L) EQYGCGTLEYLEMEDQGGAGSAGSHIKMR*NAQDELMAPASDAGYYSALTMAIFQDLGFYQ* 356

GP63_LEIAM(L) EQYGCNSLEYLEMEDQG-SAAPGSHIK*A*-*NAQDELMAPTASA*GYYTALTMAVFQDLGFYQ 366

GP63_LEIMA(L) EQYGCDTLEYLEVEDQGGAGSAGSHIKMR*NAQDELMAPAAAAGYYTALTMAIFQDLGFYQ* 369

GP63_LEIDO3(C) *EQYGCXTLEYLEIEDQGGAGSAGSHIK*MR*NAKDELMAPAAAAGYYSALTMAIFQDLGFYQ* 366

GP63_LEIDO2(C) EQYGCGTLEYLEIEDQGGAGSAGSHIKMR*NAKDELMAPAAXAGYYSALTMAIFQDLGFYQ* 366

LmjF.10.470(C) EQYGCDTLEYLEVED*QGGAGSAGSHIK*MR*NAQDELMAPAAAAGYYTALTMAIFQDLGFYQ* 369

GP63_LEIDO6(C) *EXYGCDTLEYLEIEDQGGAGSAGSHIK*MR*NAKDELMAPAAAAGYYSALTMAIFQDLGFYQ* 94

GP63_LEIDO5(C) EQYGCGTLEYLEIEDQGGAGSAGSHIKMR*NAKDELMAPAAAAGYYSALTMAIFQDLGFYX* 85

LinJ.10.510(C) *EQYGCDTLEYLEIEDQGGAGSAGSHIK*MR*NAKDELMAPAAAAGYYSALTMAIFQDLGFYQ* 366

GP63_LEIME(C) EQYGCNSLEYLEIEDQGGAGSAGSHIKMRNAKDELMAPAASAGYYTALTMAVFQDLGFYQ 371

GP63_LEITR(C) EQYGCGTLEYLEIEDQGGAGSAGSHIKMRNAQDELMAPAAAGGYYTALTMAVFQDLGFYQ 370

LmxM.10.460(C) EQYGCNSLEYLEIEDQGGAGSAGSHIKMRNAQDELMAPAASAGYYTALTMAVFQDLGFYQ 371

LinJ.28.600(C?) LHYGCK*TQASMELEDEGGK*GTVSSHWKRRSAKDELMAGFSGVGVYSALTIAAMEDTGYYQ 336

*** : :*:**:* : .** * .*:***** : * *:***:* ::* *:*

LcMSPS1 ADFSKAEVMPWGRNAGCAFLSEKCMERNITKWPAMFCNENEVTMRCPTSRLSLGKCGVTR 426

LcMSPL1 ADFSKAEVMPWGRNAGCAFLSEKCMERNITKWPAMFCNENEVTMRCPTSRLSLGKCGVTR 426

LEIDOC1 ADFSKAEVMPWGRNAGCAFLSEKCMERNITKWPAMFCNENEVTMRCPTSRLMVGTCGIR- 424

MSPC_partial ADFSKAEVMPWGRNAGCAFLSEKCMERNITKWPAMFCNENEVTMRCPTSRLMVGTCGIR- 154

LinJ.10.0520(S) *ADFSKAEVMPWGRNAGCAFLSEK*CMERNITK*WPAMFCNENEVTMR*CPTSRLSLGKCGVTR 426

LinJ.10.0530(S) *ADFSKAEVMPWGRNAGCAFLSEKCMEDGITKWPAMFCN*-*SDDALR*CPTSRLSLGACSLAT 425

GP63_LEIDO4(S) *ADFSKAEVMPWGRNAGCAFLSEK*CMERNITK*WPAMFCNENEVAMR*CPTSRLSLGKCGVTR 426

LcMSPL1(L) *ADFSKAEVMPWGRNAGCAFLSEK*CMERNITK*WPAMFCNENEVTMR*CPTSRLSLGKCGVTR 426

GP63_LEIDO(L) *ADFSK*AEEMPWGR*NAGCAFLSEKCMEDGITKWPAMFCNENEVTMR*CHTGRLSLGVCGLS- 415

GP63_LEIAM(L) ADFSKAEAMPWGR*NAACAFLSEK*CMANGITKWPAMFCNESADAIRCPTSRLGVGMCDVTP 426

GP63_LEIMA(L) *ADFSK*AEVMPWGQNAGCAFLTNKCMEQSVTQWPAMFCNESEDAIRCPTSRLSLGACGVT- 428

GP63_LEIDO3(C) *ADFSK*AEXMPWGR*NAGCAFLSEK*CMEXNITKWPAMFCNENEXTMRCPTSRLMXGTCGIR- 425

GP63_LEIDO2(C) *ADFSK*AEEMPWGR*NAGCAFLSEK*CMEQNITKWPAMFCNVSVDVVRCPTSRLMLGTCGIR- 425

LmjF.10.470(C) *ADFSK*AEVMPWGQNAGCAFLTNKCMEQNITQWPAMFCNESEDAIRCPTSRLLLGTCGIR- 428

GP63_LEIDO6(C) *ADFSK*AEEMPWGR*NAGCAFLSEK*CMEQNITKWPAMFCNVSVDVVRCPTSRLMLGTCGIR- 153

GP63_LEIDO5(C) *ADFSK*AEEMPWGR*NAGCAFLSEK*CMEQNITKWPAMFCNVSVDVVRCPTSRLMLGTCGIR- 144

LinJ.10.510(C) *ADFSKAEVMPWGRNAGCAFLSEK*CMERNITK*WPAMFCNENEVTMR*CPTSRLMVGTCGIR- 425

GP63_LEIME(C) ADFSKAEEMPWGR*NVGCAFLSEK*CMAKNVTKWPAMFCNESAATIRCPTDRLRVGTCGIT- 430

GP63_LEITR(C) ADFNKAKVMPWGR*NAGCAFLSEK*CMEQNITKWRAMFCNESEDVMRCPTSRLSLGTCGIR- 429

LmxM.10.460(C) ADFSKAEAMPWGR*NAGCAFLSEK*CMAKNVTKWPPMFCNESAATIRCPTDRLSLGTCGIT- 430

LinJ.28.600(C?) GNYAK*AEPMAYGHEVGCK*LSSERCVIKSTSQIPGMFCDAPDAPWSCTSDRRGIGRCILT- 395

.:: **: *.:*::..* : :::*: . :: ***: * :.* * * :

LcMSPS1 HPDLP--PYWQYFTDPSLAGISAFMDCCPVVEPYGDGSCAQRASEAGAPFKGFNVFSDAA 484

LcMSPL1 HPDLP--PYWQYFTDPSLAGISAFMDCCPVVEPYGDGSCAQRASEAGAPFKGFNVFSDAA 484

LEIDOC1 GYSTPFSLYWQYFTNASLG-LLAFLDYCPFVIGYSDGSCNQDASLAAGFFSAFNVFSDAA 483

MSPC_partial GYSTPFSLYWQYFTNASLGGYSPFLDYCPFVIGYSDGSCNQDASLAAGFFSAFNVFSDAA 214

LinJ.10.0520(S) *HPDLP--PYWQYFTDPSLAGISAFMDCCPVVEPYGDGSCAQRASEAGAPFKGFNVFSDAA* 484

LinJ.10.0530(S) FQSLP--PYWQYFTDPSLAGISAFMDYCPVVVPFGNG*SCAQNASKVMAAVQAFNVFSDAA* 483

GP63_LEIDO4(S) *HPDLP--PYWQYFTDPSLAGISAFMDCCPVVEPYGDGSCAQRASEAGAPFKGFNVFSDAA* 484

LcMSPL1(L) *HPDLP--PYWQYFTDPSLAGISAFMDCCPVVEPYGDGSCAQRASEAGAPFKGFNVFSDAA* 484

GP63_LEIDO(L) SSDIPLPPYWQYFTDPLLAGISAFMDYCPVVVPFGDGSCAQR*ASEAGAPFKGFNVFSDAA* 475

GP63_LEIAM(L) YQALP--PYLQYFTDPFLAGSSAFMDYCPVVVPYADGSCAQSASEADAAFKAFNVFSDAA 484

GP63_LEIMA(L) RHPG-LPPYWQYFTDPSLAGVSAFMDYCPVVVPYSDGSCTQRASE*AHASLLPFNVFSDAA* 487

GP63_LEIDO3(C) GYSTPFSLYWQYFTNASLGGYSPFLDYCPFVIGYSDGSCNQDASLAAGFFSAFNVFSDAA 485

GP63_LEIDO2(C) GYSTPFSPYWQYFTNISLGGYSPFLDYCPFVIGYGGGSCNQDASLATGFFGAFNVFSDAA 485

LmjF.10.470(C) EYELPLPRYWQYFTNASLGGYSPFLDYCPFVIDYADGSCNQDASSAEEFFT*AFNVFSDAA* 488

GP63_LEIDO6(C) GYSTPFSPYWQYFTNISLGGYSPFLDYCPFVIGYGDGSCNQDASLATGFFGAFNVFSDAA 213

GP63_LEIDO5(C) GYSTPFSPYWQYFTNISLGGYSPFLDYCPFVIGYGGGSCNQDASLATGFFGAFNVFSDAA 204

LinJ.10.510(C) GYSTPFSLYWQYFTNASLGGYSPFLDYCPFVIGYSDGSCNQDASLAAGFFS*AFNVFSDAA* 485

GP63_LEIME(C) AYNTSLATYWQYFTNASLGGYSPFLDYCPFVVGYRNGSCNQDASTTPDLLAAFNVFSEAA 490

GP63_LEITR(C) GYRPPLPRYWQYFTNASLGGYSPFMDYCPVVIGYANGSCNQDASSAAEFLAAFNVFSEAA 489

LmxM.10.460(C) AYNTSLATYWQYFTNASLGGYSPFLDYCPTVVGYRNGSCNQDASTAPDLLAAFNVFSETA 490

LinJ.28.600(C?) SYK*SNLPTYFQYFGDPRLGGPDPLMDFCPFVRAADDTMCAAK*TNALK*G*-----*SVYGVMS* 450

* *** : *. .::* ** * . * :. .*:. :

LcMSPS1 RCIDGAFRPKTSHGQIKSYAGLCANVRCDTATRTYSVQVHGGSGYANCTPGLRVELSTVS 544

LcMSPL1 RCIDGAFRPKTSHGIIKSYAGLCANVRCDTATRTYSVQVHGGSGYANCTPGLRVELSTVS 544

LEIDOC1 RCIDGAFRPKNRTAANGYYAGLCANVRCDTATRTYSVQVRGSMDYVNCTPGLRVELSTVS 543

MSPC_partial RCIDGAFRPKNRTAANGYYAGLCANVRXDTATRTYSVQVRGSMDYVNCTPGLRVELSTVS 274

LinJ.10.0520(S) *RCIDGAFRPK*TSHGIIK*SYAGLCANVRCDTATRTYSVQVHGGSGYANCTPGLRVELSTVS* 544

LinJ.10.0530(S) *RCIDGAFRPKTTETVTNSYAGLCANVRCDTATRTYSVQVRGGSGYASCTPGLRVELSTVS* 543

GP63_LEIDO4(S) *RCIDGAFRPK*TSHGIIK*SYAGLCANVRCDTATR*TYSVQVHGGSGYANCTPGLR*VELSTVS* 544

LcMSPL1(L) *RCIDGAFRPK*TSHGII*KSYAGLCANVRCDTATRTYSVQVHGGSGYANCTPGLRVELSTVS* 544

GP63_LEIDO(L) *RCIDGAFRPKTTETVTNSYAGLCANVRCDTATR*TYSVQVHGGSGYANCTPGLR*VELSTVS* 535

GP63_LEIAM(L) A*CIDGAFRPK*TTHGLIKSYAALCANVKCDTAAR*TYSVQVR*G*SSGYANCTPGLR*FDLSTVS 544

GP63_LEIMA(L) *RCIDGAFRPK*ATDGIVKSYAGLCANVQCDTATRTYSVQVHGSNDYTNCTPGLRVELSTVS 547

GP63_LEIDO3(C) R*CIDGAFRPK*NRTAANGYYAGLCANVRCDTATR*TYSVQVR*GSMDYVNCTPGLRVELSTVS 545

GP63_LEIDO2(C) R*CIDGAFRPK*NRTAADGYYAGLCANVRCDTATRTYSVQVCGSMDYVNCTPGLRVELSSVS 545

LmjF.10.470(C) *RCIDGAFRPK*ATNGIVKSYAGLCANVQCDTATRTYSVQVHGSNDYTNCTPGLRVELSTVS 548

GP63_LEIDO6(C) R*CIDGAFRPK*NRTAADGYYAGLCANVRCDTATR*TYSVQVR*GSMDYVNCTPGLR*VELSTVS* 273

GP63_LEIDO5(C) R*CIDGAFRPK*NRTAADGYYAGLCANVRCDTATRTYSVQVCGSMDYVNCTPGLRVELSSVS 264

LinJ.10.510(C) *RCIDGAFRPKNRTAANGYYAGLCANVR*CDTATR*TYSVQVR*GSMDYVNCTPGLR*VELSTVS* 545

GP63_LEIME(C) RCIDGAFTPKNRTAADGYYTALCANVKCDTATR*TYSVQVR*GTNGYANCTPGLRVKLSSVS 550

GP63_LEITR(C) RCIDGAFTPKNRTAADGYYAGLCANVRCDTATR*TYSVQVR*GSMDYVSCTPGLRVELSTVS 549

LmxM.10.460(C) RCIDGAFTPKNRTAADGYYGGLCANVKCDTATR*TYSVQVR*GSNGYVNCTPGLRVKLSSVS 550

LinJ.28.600(C?) *RCVDTPAGFSIDDSAVQQHG*-*ICAEVQCG*--*SSAYGVKINGASAFR*DCPPGSTYNLSTLS 507

*:* . . : :**:*: . : :*.*:: * : .*.** .**::*

LcMSPS1 SAFEEGGYITCPPYVEVCQGNVQAAKD--------------GGNAAAGRRGPRAAATALL 590

LcMSPL1 SAFEEGGYITCPPYVEVCQGNVQAAKD--------------GGNAAAGRRGPRAAATALL 590

LEIDOC1 SAFEEGGYITCPPYVEVCQANVKGAKDFAGDSDSSSSAGDAADRAAMQRWNDRMAGLATA 603

MSPC_partial SAFEEGGYITCPPYVEVCQANVKGAKDFAGDSDSSSSAGDAADRAAMQRWNDRMAGLATA 334

LinJ.10.0520(S) *SAFEEGGYITCPPYVEVCQGNVQAAK*D--------------GGNAAAGRRGPRAAATALL 590

LinJ.10.0530(S) *SAFEEGGYITCPPYVEVCQGNVQAAK*D--------------GGNAAAGRRGPRAAATALL 589

GP63_LEIDO4(S) *SAFEEGGYITCPPYVEVCQGNVQAAK*D--------------GGNAAAGRRGPRAAATALL 590

LcMSPL1(L) *SAFEEGGYITCPPYVEVCQGNVQAAK*D--------------GGNAAAGRRGPRAAATALL 590

GP63_LEIDO(L) *SAFEEGGYITCPPYVEVCQGNVQAAK*D--------------GGNAAAGRRGPRAAATALL 581

GP63_LEIAM(L) DAFEKGGYVTCPPYVEVCQGNAQAIKD--------------GGNAA-GRRGPR-AATALV 588

GP63_LEIMA(L) NAFEGGGYITCPPYVEVCQGNVQAAKD--------------GGNTAAGRRGPRAAATALL 593

GP63_LEIDO3(C) SAFEEGGYITCPPYVE-------------------------------------------- 561

GP63_LEIDO2(C) SAFEEGGYITCPPYVE-------------------------------------------- 561

LmjF.10.470(C) KTFEEGGYITCPPYV*EVCQGNVQAAK*DFDGDSDSSSSSSDAADKAAIERWNERMAGLATA 608

GP63_LEIDO6(C) *SAFEEGGYITCPPYVEVCQANVKGAK*DFAGDSDSSSSAGDAADRAAMQRWNDRMAGLATA 333

GP63_LEIDO5(C) SAFEEGGYITCPPYVEVCQANVKGAKDFAGDSDSSSSAGDAADRAAMQRWNDRMAGLATA 324

LinJ.10.510(C) *SAFEEGGYITCPPYVEVCQANVKGAK*DFAGDSDSSSSAGDAADRAAMQRWNDRMAGLATA 605

GP63_LEIME(C) DAFEKGGYVTCPPYVEVCQGNVKAAKDFAGDTDSSSSADDAADKEAMQRWSDRMAALATA 610

GP63_LEITR(C) NAFEEGGCITCPPYVEVCQGNVKGAKDFAGDSDSSSSADDAAGKAAMLRWNDRMVGLATA 609

LmxM.10.460(C) DAFEKGGYVTCPPYVEVCQGNVKAAKDFAGDTDSSSSADDAADKEAMQRWSDRVAALATA 610

LinJ.28.600(C) PSFSKG-HLVCPSYESVCAININASLY---------------EEYSRLLTDHSVTGARTS 551

:*. * :.**.* .

LcMSPS1 VAALLAVAL-------------------------------------- 599

LcMSPL1 VAALLAVAL-------------------------------------- 599

LEIDOC1 AMVLLGMVLSLMALVVVWLLLLTCPWWCCKFGGLPT----------- 639

MSPC_partial AMVLLGMVLSLMALVVVWLLLLTCPWWCCKFGGLPT----------- 370

LinJ.10.0520(S) VAALLAVAL-------------------------------------- 599

LinJ.10.0530(S) VAALLAVAL-------------------------------------- 598

GP63_LEIDO4(S) VAALLAVAL-------------------------------------- 599

LcMSPL1(L) VAALLAVAL-------------------------------------- 599

GP63_LEIDO(L) VAALLAVAL-------------------------------------- 590

GP63_LEIAM(L) VAALLAVAL-------------------------------------- 597

GP63_LEIMA(L) VAALLAVAL-------------------------------------- 602

GP63_LEIDO3(C) -----------------------------------------------

GP63_LEIDO2(C) -----------------------------------------------

LmjF.10.470(C) ATVLLGVVLSLMALVVVWLLLVSCPRWCCKVGGLPT----------- 644

GP63_LEIDO6(C) AMVLLGMVLSLMALVVVWLLLLTCPWWCCKFGGLPT----------- 369

GP63_LEIDO5(C) AMVLLGMVLSLMALVVVWLLLLTCPWWCCKFGGLPT----------- 360

LinJ.10.510(C) AMVLLGMVLSLMALVVVWLLLLTCPWWCCKFGGLPT----------- 641

GP63_LEIME(C) TTLLLGMVLSLMALLVVRLLLTSSPWCCCRLGG-LPT---------- 646

GP63_LEITR(C) ATVLLGMVLSLMALVVVWLLLVSCPWWCCKLGGPPASVTPACSPETE 656

LmxM.10.460(C) TTLLLGMVLSLVTLLVVRLLLTSSPWCCCRLGGGAPDVSCDGPLA-- 655

LinJ.28.600(C?) VTAVVAVLLVVLFMG-------------------------------- 566

[8]. Ramamoorthy R, Donelson JE, Paetz KE, Maybodi M, Roberts SC, Wilson ME. Three distinct RNAs for the surface protease gp63 are differentially expressed during development of *Leishmania donovani chagasi* promastigotes to an infectious form. J Biol Chem. 1992;267:1888–95.

[9]. Roberts SC, Swihart KG, Agey MW, Ramamoorthy R, Wilson ME, Donelson JE. Sequence diversity and organization of the msp gene family encoding gp63 of *Leishmania chagasi*. Mol Biochem Parasitol. 1993;62:157-71.

[24]. Yao C, Donelson JE, Wilson ME. The major surface protease (MSP or GP63) of Leishmania sp. Biosynthesis, regulation of expression, and function. Mol Biochem Parasitol. 2003;132:1–16.

[30]. Roberts SC, Wilson ME, Donelson JE. Developmentally regulated expression of a novel 59-kDa product of the major surface protease (Msp or gp63) gene family of *Leishmania chagasi*. J Biol Chem. 1995;270:8884-92.
